# Beclin 1-Mediated Autophagy Is Potentiated by an Interaction with the Neuronal Adaptor FE65

**DOI:** 10.3390/biology14010097

**Published:** 2025-01-18

**Authors:** Wai Wa Ray Chan, Jessica Chow, Dennis Dik-Long Chau, Yuqi Zhai, Kwok-Fai Lau

**Affiliations:** School of Life Sciences, Faculty of Science, The Chinese University of Hong Kong, Hong Kong, China; waiwachan1114@gmail.com (W.W.R.C.); jessicachow@link.cuhk.edu.hk (J.C.); denniscdl@link.cuhk.edu.hk (D.D.-L.C.); 1155118995@link.cuhk.edu.hk (Y.Z.)

**Keywords:** FE65, Beclin 1, macroautophagy

## Abstract

Autophagy is a vital cellular pathway in eukaryotic cells, including neurons, where it plays significant roles in neurodevelopment and maintenance. A crucial step in autophagy is the formation of the class III phosphatidylinositol 3-kinase complex 1 (PI3KC3-C1), which is essential for initiating autophagosome biogenesis. Beclin 1 is the key component of PI3KC3-C1, and its interactors have been reported to affect autophagy. The brain-enriched adaptor protein FE65 has been shown to interact with Alzheimer’s disease amyloid precursor protein (APP) to alter the processing of APP. Additionally, FE65 has been implicated in various cellular pathways, including autophagy. We demonstrate here that FE65 positively regulates autophagy. FE65, through its C-terminus, has been shown to interact with Beclin 1. Notably, the overexpression of FE65 enhances Beclin 1-mediated autophagy, whereas this process is attenuated in FE65 knockout cells. Moreover, the stimulatory effect of FE65 on Beclin 1-mediated autophagy is diminished by an FE65 C-terminus deletion mutant that disrupts the FE65–Beclin 1 interaction. Lastly, we have found that the FE65-Beclin 1 interaction modulates the kinase activity of the PI3KC3-C1 complex. Together, we have identified FE65 as a novel Beclin 1 interactor, and this interaction potentiates autophagy.

## Simple Summary

Autophagy is a “self-eating” process that is important for cells to dispose of and/or recycle any component. Beclin 1 is the crucial member of the autophagic process in cells. It acts as the key regulator as it interacts with different binding partners to modify the activity of autophagy. This article explores how FE65, a protein mainly found in the brain, enhances autophagy by interacting with Beclin 1.

## 1. Introduction

Autophagy is a highly conserved metabolic recycling system that plays a crucial role in capturing, sequestering, breaking down, and recycling proteins and cellular components in all eukaryotic cells. There are various types of autophagy, including macroautophagy, chaperone-mediated autophagy, and microautophagy, each characterized by distinct mechanisms and specific targets for degradation [1,2,3]. In this study, we will specifically focus on the cellular mechanism of macroautophagy, hereafter referred to as autophagy. Autophagy can generally be divided into five stages: nucleation, elongation, maturation, fusion, and degradation [1]. Autophagy is induced by the mammalian target of rapamycin complex 1 (mTORC1) and the AMP-activated protein kinase (AMPK) signaling pathways under conditions of stress, such as starvation and a low ATP/ADP ratio. During the nucleation step, the assembly of the class III phosphatidylinositol 3-kinase complex 1 (PI3KC3-C1) occurs, involving core subunits vacuolar protein sorting 15 (VPS15), vacuolar protein sorting 34 (VPS34), Beclin 1, and autophagy-related gene 14 (ATG14L) [4]. The PI3KC3-C1 complex facilitates the production of phosphatidylinositol 3-phosphate (PI3P) at the membranes of the endoplasmic reticulum (ER) and mitochondria. The accumulation of PI3P at the ER membrane leads to the formation of a structure known as the omegasome, which serves as the initiation site for phagophore formation [5]. The elongation of the phagophore leads to the formation of the autophagosome and is mediated by the ATG7-ATG3-ATG16 complex, which conjugates microtubule-associated protein 1A/1B-light chain 3 (LC3) to phosphatidylethanolamine (PE) through a ubiquitin-like conjugation mechanism [6]. It is important to note that the lipidated form of LC3 (LC3-II) reflects the number of autophagosomes and serves as a key indicator of autophagic activity. The maturation of autophagosomes involves the removal of LC3-II and PI3P from the autophagosomal membrane by ATG4 and PI3P phosphatases [7]. Finally, soluble *N*-ethylmaleimide-sensitive factor attachment protein receptors (SNAREs), tethers, and Rab proteins work collaboratively to facilitate the fusion of mature autophagosomes with lysosomes, enabling the degradation of cargo within the autophagosomes by lysosomal enzymes [8].

In other words, autophagy is a well-regulated system orchestrated by the formation of various functional protein complexes. The PI3KC3-C1 complex is one of the most studied complexes, with its function and structure well characterized. The four core components that form the PI3KC3-C1 complex are VPS15, VPS34, Beclin 1, and ATG14L. VPS15 comprises an N-terminal pseudo-kinase domain, a HEAT domain, and a C-terminal WD40 domain. VPS34 serves as the catalytic subunit of the complex and consists of an N-terminal lipid-associating C2 domain, a helical domain, and a kinase domain at the C-terminus, which is responsible for converting PI lipids into PI3P. Beclin 1 features a BH3 domain at its N-terminus, a coiled-coil domain, and a C-terminal Evolutionarily Conserved Domain (ECD)/β-α autophagy-specific (BARA) domain. ATG14L is a subunit unique to the autophagic PI3KC3-C1 complex, also containing a coiled-coil domain and a Barkor/Atg14L autophagosome-targeting sequence (BATS) domain, which is essential for direct membrane binding [9,10,11,12]. Structurally, the PI3KC3-C1 complex is characterized by a “V shape”, with VPS15, the largest protein in the complex, serving as a backbone to organize the other components. The N-terminal region of VPS15 is shown to associate with the helical domain of VPS34, while its WD40 propeller domain interacts with the BARA domain of Beclin 1. Beclin 1 and ATG14L are held together as their coiled-coil domains intertwine to form a dimer [4,13]. Notably, Beclin 1, VPS34, and VPS15 also form the PI3KC3-C2 complex with UV-radiation resistance-associated gene protein (UVRAG), which contributes to autophagosome maturation and endocytosis; however, the underlying mechanism remains unestablished [14,15].

Among the four core subunits, Beclin 1 is the key regulator that modulates the activity of the PI3KC3-C1 complex and, consequently, regulates autophagic activity. The multiple protein–protein interaction domains on Beclin 1 allow it to interact with different proteins, and its interactome usually regulates the activity of the PI3KC3-C1 complex through its effects on Beclin 1. For example, calcium/calmodulin-dependent protein kinase II (CaMKII) interacts with and phosphorylates the N-terminus of Beclin 1, subsequently promoting the tumor necrosis factor (TNF) receptor-associated factor 6 (TRAF6)-mediated ubiquitination of Beclin 1, which stimulates the activity of the PI3KC3-C1 complex [16,17]. Conversely, BCR-ABL1 tyrosine kinase also interacts with and phosphorylates Beclin 1, but this inhibits autophagy by disrupting the assembly of the PI3KC3-C1 complex [18]. Both B-cell leukemia/lymphoma 2 protein (Bcl-2) and mammalian Ste20-like kinase 1 (Mst1) interact with Beclin 1, inhibiting its interaction with VPS34, which suppresses the activity of the PI3KC3-C1 complex and consequently negatively regulates autophagy [19,20]. Cellular FLICE-like inhibitory protein (c-FLIP) is another interactor of Beclin 1 that positively regulates autophagy by stabilizing Beclin 1 and enhancing the activity of the PI3KC3-C1 complex [21].

FE65 is a brain-enriched adaptor protein that contains multiple protein–protein interaction domains, including an N-terminal tryptophan-tryptophan (WW) domain and two phosphotyrosine-binding (PTB) domains at the C-terminus [22]. FE65 interacts with various partners to participate in different processes. For instances, FE65 interacts with the amyloid precursor protein intracellular domain (AICD) and Tat interactive protein 60kDa histone acetyltransferase (Tip60) to regulate neurogenesis-related transactivation signaling [23]. FE65 also participates in stimulating neurite outgrowth by interacting with ADP-ribosylation factor 6 (ARF6) and Ras-related C3 botulinum toxin substrate 1 (Rac1) guanine nucleotide exchange factors (GEFs), such as Arf nucleotide binding site opener (ARNO) and engulfment and cell motility protein 1 (ELMO1), which activates ARF6 and Rac1 [24,25]. Moreover, the interaction between FE65 and APP can lead to mitochondrial dysfunction by destabilizing actin fibers in neurons, resulting in decreased mitochondrial membrane potential and reduced ATP production [26,27]. Despite studies on FE65 being primarily focused on neurodevelopment and neurodegeneration, increasing evidence suggests that FE65 may also participate in neuronal autophagy. FE65 interacts with ARF6 through its PTB1 domain, while ARF6 serves as a positive regulator of autophagy by promoting autophagosome formation [28]. Additionally, FE65 can interact with Abelson tyrosine kinase (c-Abl), whose activity has recently been shown to regulate autophagic flux [29,30].Mammalian-enabled protein (MENA) is another interactor of FE65, known to co-localize with actin and regulate actin-based cell motility [31,32]. Interestingly, MENA is found to play a crucial role in autophagosome formation via its actin regulatory function [33].

In this study, we identified FE65 as a positive regulator of autophagy. We revealed a novel interaction between FE65 and Beclin 1, demonstrating that the loss of this interaction can attenuate autophagy. Lastly, we showed that FE65 mediates autophagy through its interaction with Beclin 1, which affects the kinase activity of the PI3KC3-C1 complex.

## 2. Materials and Methods

### 2.1. Cell Cultures and Transfection

Human embryonic kidney 293 (HEK293) cells (obtained from American Type Culture Collection, ATCC), stably transfected cell lines, and COS7 cells were cultured in DMEM (Gibco, Jenks, OK, USA) supplemented with 10% *v*/*v* fetal bovine serum (FBS) at 37 °C in a 5% CO_2_ environment. Cells were transfected using polyethylenimine (PEI) (Polysciences) or EndoFectin™ Max (Genecopia) according to the manufacturers’ instructions. 48 h post-transfection, cells were harvested for analysis.

### 2.2. Plasmids

Mammalian expression of the wild-type pCI-FE65wt construct was obtained and prepared as described [25]. The pCI-FE65^ΔCt^ (amino acid 1–668) construct was generated by PCR with forward primer (5′-ATCGGTCGACATGTCTGTTCCATCATCACTGAGCCAGTC-3′) and reverse primer (5′- CGATGCGGCCGCTCACTGGGAACGGGCATCCAGA-3′). FLAG-tagged Beclin 1 was kindly gifted by Rubinsztein DC [34].

### 2.3. FE65, FE65^ΔCt^, and GFP-LC3 Stably Transfected Cell Line

pSELECT-puro-FE65, pSELECT-puro-FE65^ΔCt^, and GFP-LC3 were transfected into HEK293 cells using EndoFectin™ Max for 48 h. Following transfection, cells were selected with 3 μg/mL of puromycin for 36 h. The expression levels of FE65 and FE65^ΔCt^ in the isolated clones were confirmed and compared by Western blot analysis. The optimal expression level of GFP-LC3 in the isolated clones was validated using both Western blot and fluorescence microscopy.

### 2.4. FE65 Knock out (KO) Cell Line

FE65 KO cells were generated as previously described [35]. Briefly, single guide RNAs (sgRNAs) were designed to target FE65 at exon 2 according to the online database (https://www.idtdna.com/page, accessed on 18 July 2020). The oligo sequences were as follows: FE65 sgRNA #1_F (5′ CACCGTGTTGGCATTAATGGCCGAC 3′), FE65 sgRNA #1_R (5′ AAACGTCGGCCATTAATGCCAACAC 3′), FE65 sgRNA #2_F (5′ CACCGAAGGACCTGCGCAGCGCCAT 3′), and FE65 sgRNA #2_R (5′ AAACATGGCGCTGCGCAGGTCCTTC 3′). The oligos were then inserted into the pSpCas9(BB)-2A-Puro (PX459) vector [36]. Cells were then co-transfected with the sgRNAs for 48 h and selected with 3 μg/mL of puromycin (Invivogen, San Diego, CA, USA) for 36 h. The expression level of FE65 in the selected clones was validated by Western blot.

### 2.5. FE65 Knockdown

The knockdown of FE65 in HEK293 cells was performed as previously described [24,37]. ON-TARGET plus siRNAs (purchased from Horizon Discovery) were transfected into HEK293 cells using RNAiMAX (Invitrogen) according to the manufacturer’s instructions. ON-TARGET plus non-targeting siRNAs were used as control siRNA knockdown.

### 2.6. GFP-LC3 Cleavage and Puncta Formation Assay

GFP-LC3 cleavage and puncta formation assays were performed as previously described [38]. For the GFP-LC3 cleavage assay, plasmids expressing GFP-LC3 were transfected into empty vector-, FE65-, and FE65^ΔCt^-stable cell lines. Cells were treated with 10 μM chloroquine (CQ) for 24 h. After treatment, cells were simply resolved in SDS sample buffer (50 mM Tris-HCl pH 6.8, 10% glycerol, 2% SDS, 5% β-mercaptoethanol, 0.01% bromophenol blue). The protein content would then be analyzed by Western blot. The level of GFP-LC3 and free GFP was detected by anti-GFP antibody (rat). For the GFP-LC3 puncta formation assay, GFP-LC3-stably transfected cell lines were generated as described. The stable cells were seeded in 12-well cell culture dishes with the addition of coverslips. FE65 and FE65^ΔCt^ were transfected to the cells with EndoFectin™ Max (Genecopia, Rockville, MD, USA) according to the manufacturer’s instructions. In addition, 24 h post transfection, cells were treated with 150 nM Bafilomycin A1 (Baf A1) for 1 h. The coverslips were then fixed in 4% (*w*/*v*) paraformaldehyde (PFA) for 10 min and permeabilized with 0.1% (*v*/*v*) Triton-X. After blocking in 5% (*v*/*v*) FBS in PBS, cells were immunostained with anti-FE65 antibody (rabbit), anti-FLAG antibody (M2, Sigma, Oakville, ON, USA) and DAPI.

### 2.7. LC3 Conversion and p62 Turnover Assays

LC3 conversion and p62 turnover assays were performed as previously described [38,39,40]. Empty vector-, FE65-, and FE65^ΔCt^-stably transfected cells were seeded in 12-well cell culture dishes. Upon reaching 80% confluency, cells were treated with 150 nM Baf A1 or 100 μM CQ for 1 h or 24 h as indicated, before harvesting in SDS sample buffer and analyzed by Western blot. Assays were also performed in FE65 KO cells. The levels of LC3, p62, FE65, Beclin 1, and tubulin were detected by anti-LC3 antibody (ProteinTech, San Diego, CA, USA), anti-p62 antibody (ProteinTech), anti-FE65 antibody (E20, Santa Cruz, Dallas, TX, USA), anti-Beclin 1 antibody (rat), and anti-tubulin (DM1A, Santa Cruz).

### 2.8. Proximity Ligation Assay (PLA)

PLA was performed using the Duolink In Situ-Fluorescence kit (Sigma) as previously described [25]. In brief, HEK293 cells were seeded in a 24-well plate with coverslips. pCI-FE65wt, pCI-FE65^ΔCt^, and/or FLAG-Beclin 1 plasmids were transfected with EndoFectin™ Max (Genecopia). In addition, 24 h post-transfection, cells were fixed in 4% PFA and permeabilized in 0.1% Triton-X. After blocking in 5% FBS, cells were incubated with goat anti-FE65 antibody (E20, Santa Cruz) [1:2000] and mouse anti-FLAG antibody (M2, Sigma) [1:2000] for 1 h at room temperature. β-tubulin and the nucleus were also stained by anti-β-tubulin and DAPI, respectively, to monitor the cell morphology. According to the manufacturer’s protocol, cells were then incubated with PLA probe anti-mouse PLUS and anti-goat MINUS for 1 h in a humid 37 °C incubator. Ligation reaction was then started by adding ligase and incubated for 30 min at 37 °C. After that, polymerase was added to start the amplification for 100 min at 37 °C. Finally, the coverslips were mounted in DAKO mounting medium (Merck, Rahway, NJ, USA).

### 2.9. PI3KC3 Kinase Activity Assay

HEK293 cells were seeded in 10 cm dishes and transfected with myc-tagged ATG14L, FLAG-tagged Beclin 1, HA-tagged VPS34, and non-tagged VPS15 along with empty vector, FE65, or FE65^ΔCt^. In addition, 48 h after transfection, cells were collected and proceeded to perform the PI3KC3 kinase activity assay according to Park et al., 2016 [41]. Briefly, transfected cells were harvested in Buffer A (20 mM Tris-HCl pH7.5, 137 mM NaCl, 1 mM MgCl_2_, 1 mM CaCl_2_, 1% (*v*/*v*) NP-40, 10% (*v*/*v*) glycerol, and protease inhibitor). Clear supernatants were collected by centrifugation at 13,000× *g* for 10 min at 4 °C. The PI3KC3-C1 complex would then be immunoprecipitated by anti-myc antibody (ProteinTech) overnight at 4 °C. Immunoprecipitants were captured by Protein A agarose (Beyotime) for 1 h at 4 °C, followed by multiple washes with the subsequent buffers: PBS with 1% (*v*/*v*) NP-40, wash buffer (100 mM Tris-HCl pH 7.5 and 500 mM LiCl), TNE buffer (10 mM Tris-HCl pH 7.5, 100 mM NaCl, and 1 mM EDTA), and kinase buffer (50 mM HEPES pH 7.5, 150 mM NaCl, 1 mM CHAPS, and 5 mM MnCl_2_). The isolated PI3KC3-C1 complex would then react with 25 ug/mL of PI lipid substrate and 1 mM ATP, at 30 °C for 30 min, to produce PI3P product. The reactions were stopped by separating the reactant from the kinase and immediately proceeded to run onto the PVDF membrane using the Hybri-Dot Vacuum Manifold 1050 mm 96-well Dot Blot apparatus (Bristol Robotics Laboratory, Bristol, UK). The membrane was then blocked with 3% BSA and the level of PI3P was detected by incubating with GST-px40 (1 mg/mL) for 2 h at room temperature and anti-GST antibody (rat) overnight at 4 °C.

### 2.10. Protein–Protein Interaction Assays

In general, cells were seeded in 10 cm dishes and transfected with targeted proteins as indicated. In addition, 48 h post-transfection, cells were harvested in ice-cold lysis buffer (50 mM Tris-HCl, pH 7.5, 150 mM NaCl, 1 mM EDTA, 1% Triton X-100, 1 mM PMSF). Clear supernatants were collected by centrifugation at 150,000 rpm for 10 min at 4 °C. Cleared cell lysates were then used in further assays. In glutathione S-transferase (GST) pulldown assays, bacterial expressed and purified GST and GST-tagged proteins were captured by glutathione-Sepharose 4B (GE Healthcare Biosciences, Pittsburgh, PA, USA). The captured GST-tagged proteins were then incubated in transfected cell lysates overnight at 4 °C. Each reaction was washed with ice-cold lysis buffer three times and resuspended in SDS sample buffer and subjected to SDS-PAGE and Western blot analysis. For co-immunoprecipitation assays, the cleared cell lysates or rat brain lysates were incubated with the indicated antibody overnight at 4 °C. The immunoprecipitants were then captured by Protein A agarose (Beyotime, Shanghai, China) for 2 h at 4 °C. After three washes with ice-cold lysis buffer, the immunoprecipitants were resuspended in SDS sample buffer and analyzed in Western blot.

### 2.11. Immunostaining

COS7 cells were seeded in 24-well plate with coverslips. myc-FE65 and FLAG-Beclin 1 plasmids were transfected with EndoFectin™ Max (Genecopia). In addition, 24 h post-transfection, cells were fixed in 4% PFA and permeabilized in 0.1% Triton-X. After blocking with 5% FBS, cells were immunostained with anti-FE65 antibody (rabbit), anti-FLAG antibody (M2, Sigma) and DAPI.

WD-repeat PI3P effector protein-2 (WIPI-2) immunostaining was performed as previously described [42,43,44]. HEK293 cells and FE65 KO cells were seeded in a 12-well plate with coverslips. Cells were transfected as indicated with empty vector, FE65, or FE65^ΔCt^ plasmids for 24 h using EndoFectin™ Max (Genecopia). Starvation was induced by incubating cells in Earle’s balanced salt solution (EBSS) for 2 h before fixation. After blocking with 5% FBS, endogenous WIPI-2 was immunostained with anti-WIPI-2 (Thermofisher, Waltham, MA, USA), anti-FE65 antibody (rabbit), and DAPI.

### 2.12. Statistical Analysis

Experiments were repeated at least three times. Statistical analyses were performed by one-way ANOVA with Turkey’s multiple comparison tests or unpaired *t*-tests. Statistical significance is indicated as *** *p* < 0.001; ** *p* < 0.01; * *p* < 0.05; n.s., not significant (*p* > 0.05). Error bars are shown as either SD or SEM.

## 3. Results

### 3.1. FE65 Positively Regulates Autophagy

Autophagy assays commonly rely on the detection of LC3 through techniques such as immunoblotting and immunofluorescence imaging. In this study, we employed a GFP-LC3 cleavage assay as the initial approach to validate autophagic activity. This assay exploits the differential resistance of GFP and LC3 to autophagolysosomal degradation. Briefly, during the maturation and fusion stages, autophagosomes undergo acidification to facilitate lysosomal degradation [38,45]. LC3 is rapidly degraded under acidic conditions, while GFP degradation is delayed, allowing for the detection of free GFP via Western blotting. The level of free GFP is positively correlated with autophagic activity.

**Figure 1:**
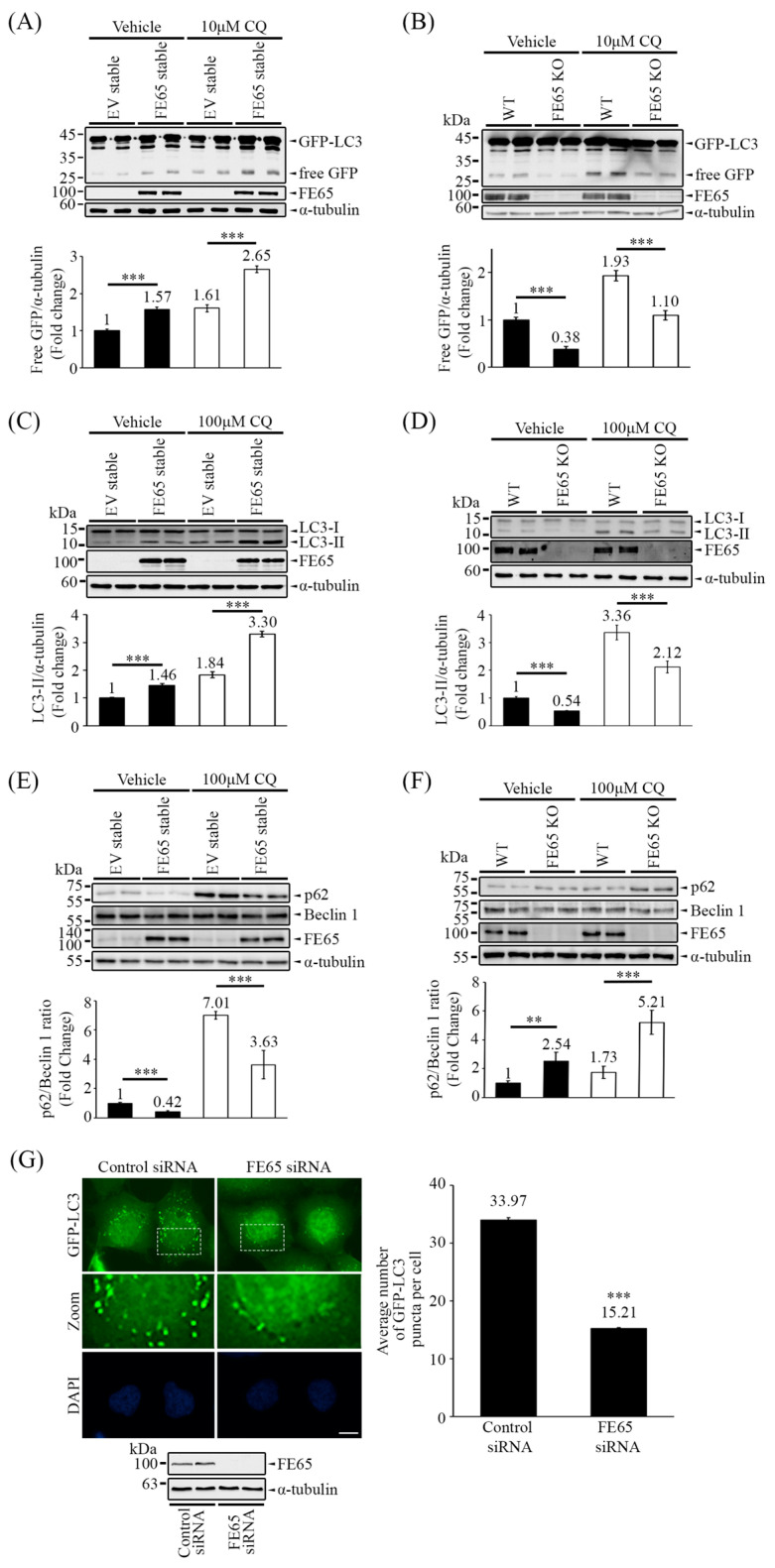
FE65 enhances autophagic activity. (**A**) The overexpression of FE65 in stable cell lines increased the level of free GFP from the cleavage of GFP-LC3, with or without CQ treatment. (**B**) The knockout of FE65 in cells attenuated the release of free GFP from GFP-LC3, again with or without CQ treatment. GFP-LC3 and GFP were detected in Western blots using an anti-GFP antibody, while the expression of FE65 was confirmed with an anti-FE65 antibody (E20). (**A**,**B**) The bar charts show the levels of free GFP; error bars represent standard deviation (SD), *** *p* < 0.001. (**C**) FE65 overexpressing stable cells exhibited increased lipidation of LC3 when treated with or without CQ. Endogenous LC3 was detected by immunoblotting using an anti-LC3 (Proteintech) antibody. (**D**) WT HEK293 and FE65 KO cells were treated with or without CQ. The knockout of FE65 decreased the level of LC3-II. (**C**,**D**) The bar charts represent the levels of LC3-II; error bars are shown as SD, *** *p* < 0.001. (**E**) Stable cells overexpressing FE65 showed a decrease in the p62/Beclin 1 ratio, regardless of CQ treatment. Endogenous p62 was detected by immunoblotting using an anti-p62 (Thermofisher) antibody. (**F**) WT HEK293 and FE65 KO cells were treated with or without CQ. The knockout of FE65 resulted in increased p62 accumulation. (**E**,**F**) The bar charts illustrate the ratio of p62 to Beclin 1, with error bars representing SD, *** *p* < 0.001, ** *p* < 0.01. (**G**) GFP-LC3 stably expressing cells were transfected with control and FE65 siRNA. GFP-LC3 puncta were counted after starvation treatment with serum-free medium for 3 h. The bar chart shows the number of puncta per cell. Data were obtained from at least 70 cells per transfection, and the experiment was repeated at least three times. Error bars are SEM, *** *p* < 0.001. Scale bar, 10 μm. Western blotting also confirmed the knockdown of FE65 in the stable cell line.

In Figure 1A,B, we observed that overexpression of FE65 in cells led to an increase in the release of free GFP from GFP-LC3, whereas knockout of FE65 resulted in a decrease in free GFP release, indicating a positive role for FE65 in autophagy. Additionally, treatment with CQ, which inhibits autophagosome acidification, led to the accumulation of free GFP. In the presence of CQ, a similar phenomenon regarding the release of free GFP is observed in Figure 1A,B, suggesting that FE65-mediated GFP-LC3 cleavage occurs at an early stage of autophagy.

To further investigate autophagic activity in mammalian cells, we employed an LC3 conversion assay. As mentioned earlier, LC3 undergoes a conjugation process and is lipidated to form LC3-II during the elongation stage of autophagy. In Figure 1C, we observed that overexpression of FE65 increased the levels of LC3-II in cells, while knockout of FE65 decreased it (Figure 1D). Additionally, we monitored the ratio of p62/Beclin 1 as the alternative readout of autophagy [38,46]. An increase in p62 levels is not only an indicator of impaired autophagy but also a sign of proteasome activity inhibition [47]. Conversely, a decrease in p62 levels and an increase in Beclin 1 are both indicators of enhanced autophagy, making the p62/Beclin 1 ratio a useful measure in this context [38,46]. Western blot analyses revealed a decrease in the p62/Beclin 1 ratio in FE65 stably transfected cells (Figure 1E). In contrast, the level of p62 was accumulated in FE65 KO cells (Figure 1F). Importantly, CQ treatment did not attenuate FE65-mediated LC3 lipidation and p62 accumulation (Figure 1C–F), suggesting that FE65 regulates autophagy at an early stage, possibly during the nucleation phase.

Figure 1G and Figure S1 demonstrates the impact of FE65 knockdown under autophagy-inducing conditions, where we counted GFP-LC3 puncta in comparison to mock knockdown cells. FE65 knockdown resulted in a decreased number of GFP-LC3 puncta, indicating impaired autophagosome formation. Overall, our data provide insights into the influence of FE65 on autophagic activity in cells, suggesting a potential regulatory role for FE65 in the autophagy process.

### 3.2. FE65 Is a Novel Interactor to Beclin 1

**Figure 2:**
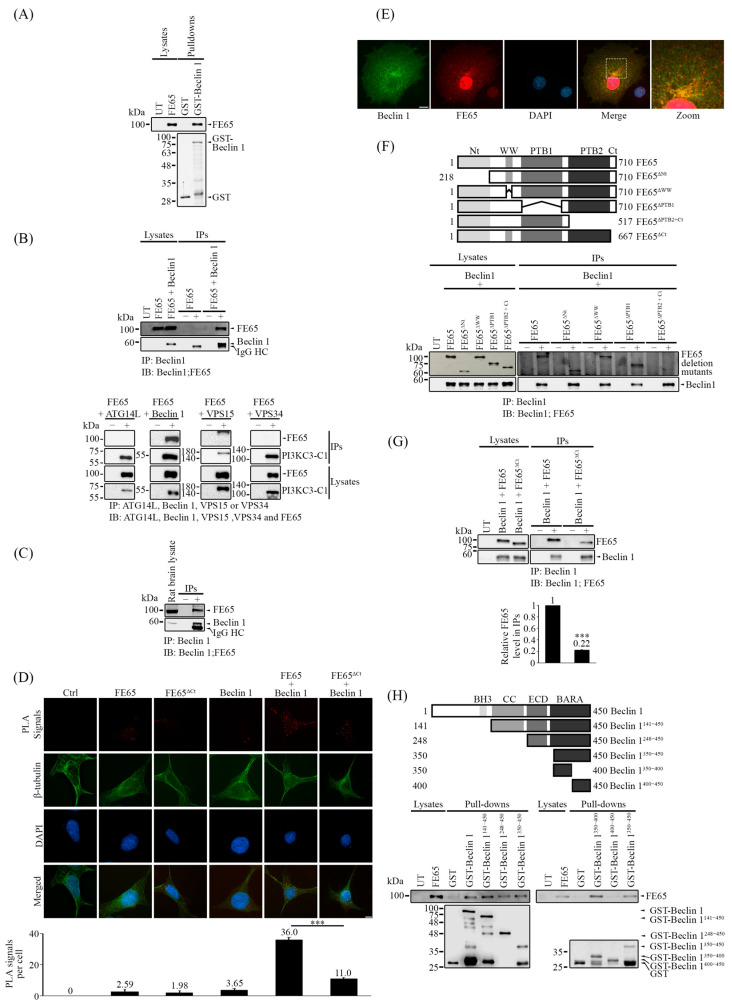
FE65 is a novel Beclin 1 interactor. (**A**–**D**) FE65 interacts with Beclin 1. (**A**) Bacterial-expressed GST and GST-Beclin 1 were used as bait to pulldown FE65 in transfected cell lysates. FE65 was specifically pulled down by GST-Beclin 1, with the amount of bait used in each pulldown shown in a Coomassie blue-stained gel. (**B**) Upper panel: HEK293 cells were transfected with either FE65 alone or FLAG-Beclin 1. Beclin 1 was immunoprecipitated using (−) a control mouse IgG or (+) an anti-FLAG antibody (M2, Sigma). The co-immunoprecipitant was detected using an anti-FE65 antibody (E20, Santa Cruz). Lower panel: HEK293 cells were transfected with myc-FE65 + FLAG-ATG14L, myc-FE65 + FLAG-Beclin 1, myc-FE65 + FLAG-VPS15, and myc-FE65 + HA-VPS34. The PI3KC3-C1 components were immunoprecipitated using either (−) control mouse IgG or (+) anti-FLAG antibody (M2, Sigma) or anti-HA antibody (12CA5, Roche). The level of FE65 in immunoprecipitate was detected using an anti-myc antibody (9B11, Cell Signaling Technology). (**C**) FE65 interacts with Beclin 1 endogenously in rat brain lysate. Beclin 1 in rat brain lysate was immunoprecipitated using an anti-Beclin 1 antibody (Proteintech), and the co-immunoprecipitant of FE65 was detected by an anti-FE65 antibody (E20, Santa Cruz). (**D**) The interaction of FE65 and Beclin 1 was confirmed in cellulo by proximity ligation assay (PLA). HEK293 cells were transfected with FE65 or FE65^ΔCt^ alone, FLAG-Beclin 1 alone, and FE65 or FE65^ΔCt^ + FLAG-Beclin 1. Goat anti-FE65 antibody (E20, Santa Cruz) [1:2000] and mouse anti-FLAG antibody (M2, Sigma) [1:2000] were used. Cells co-transfected with both FE65 and Beclin 1 exhibited an increase in PLA signals, demonstrating the interaction of FE65-Beclin1 in cellulo. PLA signals were decreased significantly in FE65^ΔCt^ + FLAG-Beclin 1 co-transfected cells, indicating an impaired interaction. The bar chart shows the PLA signals per cell. Data were obtained from at least 40 cells per transfection. The experiment was repeated at least three times. Error bars represent SEM, *** *p* < 0.001. Scale bar, 10 μm. (**E**) Immunostaining images of COS7 cells for FLAG-Beclin 1 and myc-FE65. FLAG-Beclin 1 and myc-FE65 were stained with anti-FLAG antibody (M2, Sigma) and anti-FE65 antiserum (rabbit), respectively. Nuclei were stained with DAPI. An overlapped image is shown. Scale bar, 10 μm. (**F**) The C-terminal region of FE65, containing PTB2 and the C-terminus, is responsible for interacting with Beclin 1. Constructs of different FE65 deletion mutants were generated, as shown in the upper schematic diagrams. FE65 deletion mutants were co-transfected with FLAG-Beclin 1 in HEK293 cells. FLAG-Beclin 1 was immunoprecipitated with (−) control mouse IgG or (+) an anti-FLAG antibody (M2, Sigma), and the immunoprecipitant of FE65 was detected using an anti-myc antibody (Proteintech). Only the deletion of PTB2 and the C-terminus was able to disrupt the FE65-Beclin 1 interaction. (**G**) FE65^ΔCt^ significantly impaired the FE65-Beclin 1 interaction. Co-immunoprecipitation assays were performed with FLAG-Beclin 1 co-transfected with either FE65 or FE65^ΔCt^. (−) control mouse IgG or (+) an anti-FLAG antibody (M2, Sigma) was used to immunoprecipitate FLAG-Beclin 1, and the level of FE65 in the immunoprecipitate was detected using an anti-FE65 antibody (E20, Santa Cruz). The level of FE65 that was immunoprecipitated is shown as a bar chart; error bars represent SD, *** *p* < 0.001. (**H**) Beclin 1^350–400^ is responsible for interacting with FE65. GST-tagged constructs of Beclin 1 fragments were generated, as shown in the upper schematic diagrams. GST-Beclin 1 fragments were used as bait to pulldown FE65 in transfected cell lysates. All Beclin 1 fragments containing amino acids 350–400 were able to pulldown FE65; only the fragment Beclin 1^400–450^ showed an inability to pull down FE65, indicating that the Beclin 1^350–400^ fragment is responsible for the interaction with FE65. In (**A**–**C**,**E**–**G**), 1% of cell/tissue lysates were loaded as size controls.

Based on our results, it is possible that FE65 regulates autophagy at the nucleation stage. As previously mentioned, the PI3KC3-C1 complex is essential for the nucleation stage and is assembled from VPS15, VPS34, Beclin 1, and ATG14L [4]. We discovered that FE65 interacts with Beclin 1. A bacterial GST pulldown assay was first performed, where GST and GST-Beclin 1 were expressed in *E. coli*. These proteins were then used as bait to pull down FE65 from transfected cell lysates. As shown in Figure 2A, FE65 was specifically pulled down by GST-Beclin 1. Furthermore, in a co-immunoprecipitation assay, FLAG-Beclin 1 was immunoprecipitated from lysates of cells transfected with either FE65 or FE65 and Beclin 1 together. FE65 was detected as a co-immunoprecipitant along with Beclin 1 (Figure 2B upper panel). To test if FE65 interacts with other components in the PI3KC3-C1 complex, we performed separate co-immunoprecipitation assays of FE65-ATG14L, FE65-Beclin 1, FE65-VPS15, and FE65-VPS34. As shown in Figure 2B (lower panel), FE65 was only co-immunoprecipitated with Beclin 1 but not with other components of the PI3KC3-C1 complex. The interaction between FE65 and Beclin 1 was also confirmed in rat brain lysate using co-immunoprecipitation (Figure 2C).

To verify the FE65-Beclin 1 interaction in celullo, we employed proximity ligation assay (PLA). HEK293 cells transfected with both FE65 and FLAG-Beclin 1 showed a significant increase in PLA signals compared to cells transfected with either FE65 or FLAG-Beclin 1 alone. (Figure 2D and Figure S8A). Moreover, FE65 and Beclin 1 partially colocalize in the cytoplasm (Figure 2E). Together, our findings suggest that the FE65-Beclin 1 bipartite interaction occurs intracellularly.

We then aimed to characterize this interaction. To achieve this, we generated different myc-tagged FE65 deletion constructs, including FE65^ΔNt^ (deleting amino acids 1–218), FE65^ΔWW^ (deleting amino acids 248–290), FE65^ΔPTB1^ (deleting amino acids 368–530), FE65^ΔPTB2+Ct^ (deleting amino acids 518–710), and FE65^ΔCt^ (deleting amino acids 668–710). Wild-type FE65 and its deletion mutants were co-transfected with FLAG-Beclin 1, followed by co-immunoprecipitation assays. We found that only the deletion of both the PTB2 and C-terminal regions abolished the interaction with Beclin 1 (Figure 2F). Furthermore, as shown in Figure 2D (please also see Supplementary Figure S8A) and Figure 2G, the deletion of the C-terminal region (FE65^ΔCt^) significantly impaired FE65-Beclin 1 interaction in PLA and co-immunoprecipitation assays, indicating that the C-terminal of FE65 is important for this interaction.

On the other hand, GST-Beclin 1 constructs were generated, as illustrated in the schematic diagram in Figure 2H: Beclin 1^141–450^ (which lacks the BH3 domain), Beclin 1^248–450^ (which lacks both the BH3 and coiled-coil (CC) domains), Beclin 1^141–450^ (which lacks the BH3, CC, and ECD), and Beclin 1^350–450^ (which consists of the BARA domain). Using these constructs as GST baits, all were able to pull down FE65 from transfected lysates (Figure 2H). We then divided the BARA domain in half, creating two GST-Beclin 1 constructs: Beclin 1^350–400^ and Beclin 1^400–450^. This confirmed that the 100 amino acids within the BARA domain (amino acids 350–400) are responsible for the interaction with FE65 (Figure 2H). A model by Alphafold [48,49] predicts potential interactions may occur between Beclin 1^386–390^ and FE65^680–684^, which is consistent with our domain mapping results (Figure 2G,H and Figure S2). Thus, our results indicate that the C-terminal of FE65 and a small section of the BARA domain (amino acids 350–400) of Beclin 1 are responsible for the FE65-Beclin 1 interaction.

### 3.3. The Loss of Beclin 1 Interaction with the FE65 Mutant Has Attenuated Beclin 1-Mediated Autophagy

**Figure 3:**
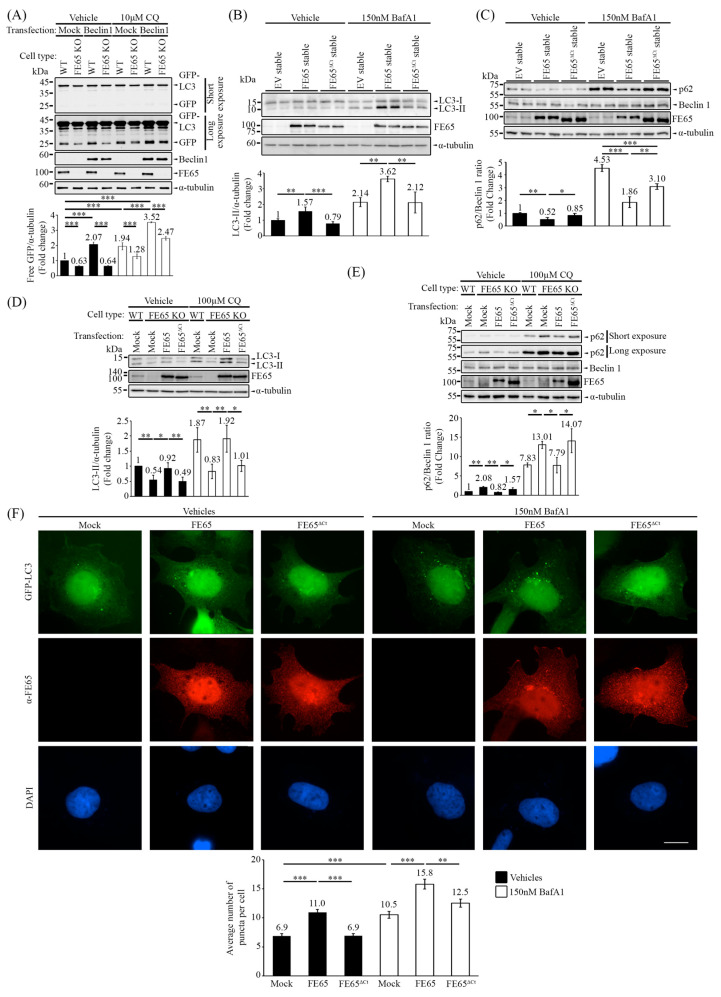
FE65-Beclin 1 interaction is essential to facilitate Beclin 1-mediated autophagy. (**A**) A GFP-LC3 cleavage assay was performed in WT HEK293 and FE65 KO cells, with and without CQ treatment. The knockout of FE65 decreased the level of free GFP from GFP-LC3 cleavage, and the overexpression of Beclin 1 failed to potentiate GFP-LC3 cleavage in FE65 KO cells. The bar chart shows the level of GFP; error bars represent standard deviation (SD), *** *p* < 0.001. (**B**) FE65, but not FE65^ΔCt^, increased endogenous LC3 lipidation in stably expressing cells, with and without Baf A1 treatment. The bar chart represents the level of LC3-II; error bars are shown as SD, *** *p* < 0.001, ** *p* < 0.01. (**C**) FE65-stably transfected cells showed a decrease in the p62/Beclin 1 ratio with and without Baf A1 treatment, whereas FE65^ΔCt^ did not exhibit this decrease. The bar chart represents the ratio of p62/Beclin 1, with error bars indicating SD. *** *p* < 0.001, ** *p* < 0.01, * *p* < 0.05. (**D**,**E**) FE65 and FE65^ΔCt^ were transfected in FE65 KO cells to assess autophagic activity. (**D**) Only FE65 was able to rescue the lipidation of LC3, regardless of CQ treatment. The bar chart illustrates the level of LC3-II; error bars are shown as SD, ** *p* < 0.01, * *p* < 0.05. (**E**) FE65 transfection in FE65 KO cells led to a significant decrease in the p62/Beclin 1 ratio, both with and without CQ treatment, while transfection with FE65^ΔCt^ attenuated this effect. The bar chart represents the ratio of p62/Beclin 1, with error bars indicating SD. ** *p* < 0.01, * *p* < 0.05. (**F**) FE65 and FE65^ΔCt^ were transfected into GFP-LC3 stably expressing cells, with and without Baf A1 treatment. GFP-LC3 puncta were counted, and overexpression of FE65 increased the number of puncta per cell, whereas overexpression of FE65^ΔCt^ failed to increase GFP-LC3 puncta. The bar chart shows the average number of puncta in cells; data were obtained from at least 40 cells, and the experiments were repeated at least 3 times. Error bars are SEM, *** *p* < 0.001, ** *p* < 0.01. Scale bar, 10 μm.

Next, we aimed to reveal how the interaction between FE65 and Beclin 1 affects autophagy. The GFP-LC3 cleavage assay was again performed in both HEK293 and FE65 KO cells. The results consistently showed that the absence of FE65 reduced the level of free GFP compared to WT HEK293 cells, both in the presence and absence of CQ, which inhibits autophagosome degradation. Interestingly, transient overexpression of Beclin 1 increased the level of free GFP in WT HEK293 cells but failed to enhance GFP-LC3 cleavage in FE65 KO cells (Figure 3A).

Having characterized the FE65-Beclin 1 interaction, we utilized FE65^ΔCt^ as a binding-defective mutant to observe how impaired FE65-Beclin 1 interaction affects autophagy. In Figure 3F and Figure S3, FE65 WT and FE65^ΔCt^ were transfected into cells stably expressing GFP-LC3. Under basal conditions, GFP-LC3 formed puncta representing autophagosome formation. The overexpression of FE65 significantly increased the number of GFP-LC3 puncta, whereas overexpression of FE65^ΔCt^ failed to do so. Treatment with Baf A1, which blocks autophagosome-lysosome fusion and leads to autophagosome accumulation, further validated that FE65 promotes autophagosome formation, while FE65^ΔCt^ attenuates this effect.

We also examined the levels of endogenous LC3 and p62 in cells stably expressing FE65 and FE65^ΔCt^ (Figure 3B,C). In Figure 3B, the overexpression of FE65 consistently increased LC3 lipidation, as indicated by the level of LC3-II, while FE65^ΔCt^ attenuated the level of LC3-II. Again, treatment with Baf A1 supported these observations. Conversely, in Figure 3C, the overexpression of FE65 triggered the decrease in the p62/Beclin 1 ratio, whereas FE65^ΔCt^ failed to do so. In addition, we overexpressed FE65 and FE65^ΔCt^ in FE65 KO cells to validate whether FE65 can rescue the autophagic activity. Our results demonstrated that the transient expression of FE65 can restore the autophagy impairment in FE65 KO cells, with or without CQ treatment, while FE65^ΔCt^ cannot (Figure 3D,E).

Taken together, the data suggest that the interaction between FE65 and Beclin 1 can potentiate autophagic activity. The loss of Beclin 1 interaction attenuates autophagy, as evidenced by reduced autophagosome formation, impaired LC3 lipidation, and increased p62/Beclin 1 ratio.

### 3.4. The Interaction of FE65-Beclin 1 Upregulates PI3KC3 Complex I Activity

Based on the previous results, it has been observed that FE65 interacts with the BARA domain of Beclin 1. Since Beclin 1 acts as the key regulator of the PI3KC3-C1 complex, we suspected that FE65 upregulates autophagy by affecting the activity of this complex. A PI3KC3-C1 activity assay was conducted to examine the impact of FE65 on PI3KC3-C1 activity through its interaction with Beclin 1.

**Figure 4:**
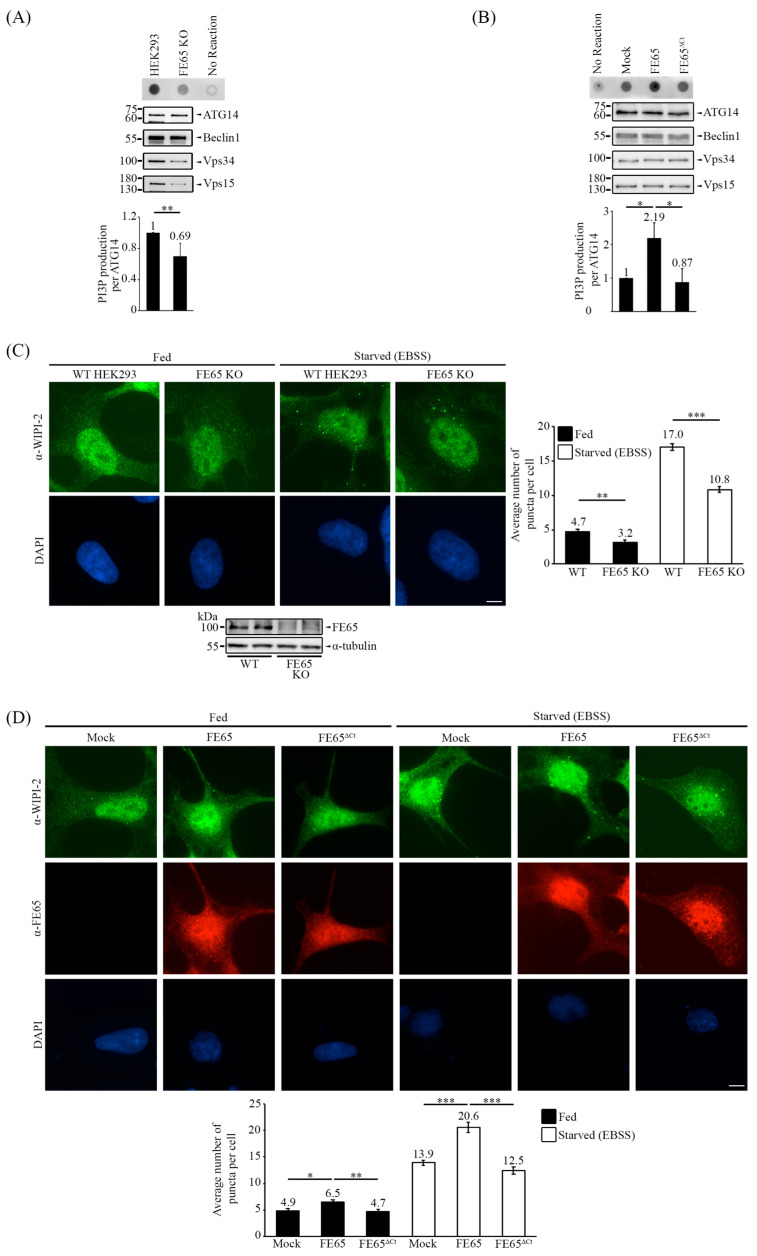
FE65 regulates autophagy by promoting PI3KC3-C1 activity. (**A**) The knockout of FE65 decreased the kinase activity of PI3KC3-C1, as indicated by a reduction in the production of PI3P. (**B**) Overexpression of FE65, but not FE65^ΔCt^, increased PI3KC3-C1 kinase activity. (**A**,**B**) The core subunits of PI3KC3-C1 were transfected into cells, and the complex was isolated using an anti-myc antibody (Proteintech). The isolated complex was incubated with PI lipid substrate and ATP. The level of PI3P was detected using dot blot analysis. The bar chart shows the level of PI3P; error bars are shown as SD, ** *p* < 0.01, * *p* < 0.05. (**C**) Immunostaining of WIPI-2 in WT HEK293 and FE65 KO cells, with and without EBSS treatment. WIPI-2 puncta were counted; the knockout of FE65 in cells has decreased the number of WIPI-2 puncta per cell. (**D**) Immunostaining of WIPI-2 in FE65 and FE65^ΔCt^ transfected cells, with and without EBSS treatment. The transient overexpression of FE65 has significantly increased the WIPI-2 puncta formation in cells, while the transient overexpression of FE65^ΔCt^ failed to do so. (**C**,**D**) The bar chart shows the average number of puncta in cells; data were obtained from at least 40 cells, and the experiments were repeated at least 3 times. Error bars are SEM, *** *p* < 0.001, ** *p* < 0.01, * *p* < 0.05. Scale bar, 10 μm.

The four components of PI3KC3-C1 (ATG14L, Beclin 1, VPS15, and VPS34) were transfected and immunoprecipitated in both WT HEK293 and FE65 KO cells. The complex was then incubated with PI lipid and ATP, and the production of PI3P was detected. The knockout of FE65 resulted in a decrease in the level of produced PI3P, indicating impaired PI3KC3-C1 kinase activity (Figure 4A). Conversely, co-transfection of the complex with FE65 increased the level of produced PI3P, while co-transfection with FE65^ΔCt^ showed a reduction in PI3P production (Figure 4B).

WIPI (WD-repeat PI3P effector protein)-2 is a PI3P effector that is recruited when autophagy is induced [38,42,50]. In order to validate whether FE65 upregulates PI3KC3 complex I activity in cellulo, we analyzed the number of WIPI-2 puncta in cells. As shown in Figure 4C (please also see the puncta count in Supplementary Figure S4 and immunofluorescence images in Figure S8B), the count of WIPI-2 puncta was significantly higher in WT HEK293 cells compared to FE65 KO cells, both with and without EBSS-induced starvation treatment. On the other hand, FE65 stimulated WIPI-2 puncta formation. However, such stimulatory effect was absent in FE65^ΔCt^ overexpressing cells (Figure 4D, Figures S5 and S8C).

These findings suggest that the FE65-Beclin 1 interaction positively regulates PI3KC3-C1 kinase activity. In summary, the interaction between FE65 and Beclin 1 has been found to upregulate autophagy by promoting PI3KC3-C1 kinase activity.

## 4. Discussion

Here, we have reported that FE65 can potentiate autophagy via its interaction with Beclin 1. Among the FE65 protein family, FE65, FE65-like 1 (FE65L1), and FE65-like 2 (FE65L2) are encoded by different genes [51]. Structurally, all FE65 proteins exhibit similar characteristics, as they each comprise a WW domain at the N-terminus and two PTB domains at the C-terminus. However, the three paralogs diverge in their N- and C-terminal regions [52]. All FE65 proteins, as adaptor proteins, interact with various binding partners and participate in diverse cellular pathways. Compared to the well-characterized and conserved protein–protein interaction domains, the C-terminal region of FE65 is less discussed. Our data suggest that the C-terminal region of FE65 is crucial for the FE65-Beclin 1 interaction, highlighting the unique role of FE65 as the sole Beclin 1 interactor and autophagy regulator among its protein family. Additionally, FE65 is primarily expressed in the nervous system, while FE65L1 and FE65L2 are expressed more broadly [53]. This suggests that FE65-mediated autophagy may predominantly occur in the nervous system. Notably, neuronal autophagy is essential for memory formation, and the selective downregulation of autophagic proteins in the hippocampus, including Beclin 1, VPS34, and VPS15, has been shown to cause memory deficits [54]. This phenomenon resembles the memory deficits observed in FE65 single knockout mice, connecting the role of FE65-mediated autophagy with autophagy-associated memory formation [55].

Impaired autophagic flux is associated with various diseases, including diabetes, cardiac hypertrophy, and autoimmune diseases [56,57,58,59]. Metformin, rapamycin, and trehalose have been studied and used as treatments, as they often improve symptoms. When examining their mechanisms of action, these drugs have been found to be autophagy enhancers. Therefore, autophagy enhancers are now considered therapeutic strategies to tackle and ameliorate these diseases [60,61]. Most of these autophagy enhancers upregulate autophagy by regulating the mTOR and/or AMPK signaling pathways. However, both mTOR and AMPK are central regulators that control not only autophagy but also cell survival, cytoskeletal organization, and other cellular functions. It is important to note that targeting mTOR or AMPK to enhance autophagy may lead to broad side effects in the human body. For example, metformin has been shown to inhibit follicle-stimulating hormone [62]. As mentioned, the interactome of Beclin 1 is crucial for regulating autophagy. Our study has demonstrated that FE65 is a novel interactor of Beclin 1 and can enhance autophagy by increasing PI3KC3-C1 activity. This finding provides new insights into how we can more specifically control autophagic flux by manipulating the Beclin 1 interactome.

Although it has been well-studied that FE65 can interact with APP and promote its processing by regulating its endosomal trafficking, the precise mechanism remains unclear. APP can be seen localized in PI3P-rich and VPS34-rich endosomes, and the decrease in PI3P in the brain perturbs APP endosomal trafficking [63]. Here, we reported that FE65 is responsible for regulating PI3P production, suggesting FE65 may associate in APP trafficking by promoting the production of PI3P. Furthermore, Yu et al. (2005) demonstrated the co-localization of APP and its proteolytic products within the autophagic vacuoles [64]. Moreover, starvation-induced autophagy has been shown to promote Aβ secretion [65,66], validating autophagosomes as the sites for amyloidogenesis. However, it remains largely unknown how APP is introduced into autophagosomes. Among the PI3KC3-C1 complex, Beclin 1 is responsible for sorting substrates into autophagosomes [67]. Notably, in the present study, we illustrate that the C-terminal region of FE65 interacts with Beclin 1, thereby increasing PI3KC3-C1 activity. FE65, as a well-established APP interactor, interacts with APP via its PTB2 domain. This may suggest FE65 acting as a bridge between Beclin 1 and APP during the autophagy nucleation stage, bringing APP into close proximity to the omegasome. This interaction may encourage the sorting of APP into the autophagosome, thereby enhancing autophagic-mediated APP processing. Altogether, our findings provide an insight into FE65 in regulating APP endosomal and autophagosomal trafficking.

In addition to facilitating the clearance of cellular constituents via lysosomal degradation, autophagy is also implicated in modulating DNA repair. Research has shown that autophagy-deficient cells are more vulnerable to DNA damage, as the loss of autophagy leads to the degradation of the DNA repair protein checkpoint kinase 1 (Chk1) [68]. Furthermore, the use of PI3K inhibitors can increase DNA damage in cancer cells, highlighting the role of autophagy in the DNA repair pathway [69]. Similarly, Beclin 1 can translocate to the nucleus and promote DNA repair following radiation-mediated DNA damage [70]. Interestingly, FE65 also influences DNA repair by translocating to the nucleus to promote the transcriptional activation of DNA damage-responsive genes [71,72,73]. Our findings broaden the connection between DNA repair and autophagy, suggesting that the role of FE65 in DNA repair may be mediated by increased autophagic flux.

## 5. Conclusions

In summary, our study reveals the interaction of FE65 and Beclin 1 and highlights the novel role of FE65 in regulating autophagy. This interaction not only widens our understanding in autophagy but also opens a new avenue for investigating the new roles of FE65.

## Data Availability

Data are contained within the article and supplementary materials.

## Supplementary Materials

The following supporting information can be downloaded at https://www.mdpi.com/article/10.3390/biology14010097/s1, Figure S1: The quantification of GFP-LC3 puncta in FE65 knockdown GFP-LC3 stably expressing cells under starvation (Figure 1G). Figure S2: The Alphafold prediction conducted using ChimeraX. Figure S3: The quantification of GFP-LC3 puncta in GFP-LC3 stably expressing cells in the presence of FE65 or FE65^ΔCt^ transfection, with and without Baf A1 treatment (Figure 3B). Figure S4: The quantification of WIPI-2 puncta in WT HEK293 and FE65 KO cells, with and without EBSS treatment (Figure 4C). Figure S5: The quantification of WIPI-2 puncta in WT HEK293 in the presence of FE65 or FE65^ΔCt^ transfection, with and without EBSS treatment (Figure 4D). Figure S6: Biological replicate images of the co-IP experiments presented in Figure 2. Figure S7: Biological replicate images of the p62 turnover experiments. Figure S8: Enlarged immunofluorescence images presented in Figure 2D and Figure 4C,D.

## Abbreviations

AICD	Amyloid Precursor Protein Intracellular Domain
AMPK	AMP-Activated Protein Kinase
APP	Amyloid Precursor Protein
ARF6	ADP-Ribosylation Factor 6
ARNO	Arf Nucleotide Binding Site Opener
ATG14L	Autophagy-Related Gene 14
Baf A1	Bafilomycin A1
BARA domain	β-α Autophagy-Specific Domain
BATS	Barkor/Atg14L Autophagosome-Targeting Sequence
Bcl-2	B-Cell Leukemia/Lymphoma 2 Protein
c-Abl	Abelson Tyrosine Kinase
c-FLIP	Cellular Flice-Like Inhibitory Protein
CaMKII	Calcium/Calmodulin-Dependent Protein Kinase II
Chk1	Checkpoint Kinase 1
CQ	Chloroquine
EBSS	Earle’s Balanced Salt Solution
ECD	Evolutionarily Conserved Domain
ELMO1	Engulfment and Cell Motility Protein 1
ER	Endoplasmic Reticulum
GEF	Guanine Nucleotide Exchange Factor
GST	Glutathione S-Transferase
HEK293	Human Embryonic Kidney 293
LC3	Microtubule-Associated Protein 1A/1B-Light Chain 3
MENA	Mammalian-Enabled Protein
Mst1	Mammalian Ste20-Like Kinase 1
mTORC1	Mammalian Target of Rapamycin Complex 1
PE	Phosphatidylethanolamine
PI3KC3-C1	Class III Phosphatidylinositol 3-Kinase Complex 1
PI3P	Phosphatidylinositol 3-Phosphate
PLA	Proximity Ligation Assay
PTB domain	Phosphotyrosine-Binding Domain
Rac1	Ras-Related C3 Botulinum Toxin Substrate 1
SNAREs	Soluble N-ethylmaleimide-Sensitive Factor Attachment Protein Receptors
Tip60	Tat Interactive Protein 60 kDa Histone Acetyltransferase
TRAF6	Tumor Necrosis Factor Receptor-Associated Factor 6
UVRAG	UV-Radiation Resistance-Associated Gene Protein
VPS15	Vacuolar Protein Sorting 15
VPS34	Vacuolar Protein Sorting 34
WIPI-2	WD-repeat PI3P effector protein-2
WW domain	Tryptophan-Tryptophan Domain
