# Supplementary material for: Beclin 1-Mediated Autophagy Is Potentiated by an Interaction with the Neuronal Adaptor FE65
Source: Biology (Basel). 2025 Jan 18;14(1):97. doi: 10.3390/biology14010097 (PMC11763304; doi:10.3390/biology14010097)

Figure S1. The quantification of GFP-LC3 puncta in FE65 knockdown GFP-LC3 stably expressing cells under starvation (Figure 1G). The experiments were performed in three independent replicates. In each experiment, at least 40 cells were analyzed per transfection. Data from experiment 1 (boxed in green) is presented in Figure 1E. Error bars are SEM, *** p < 0.001.

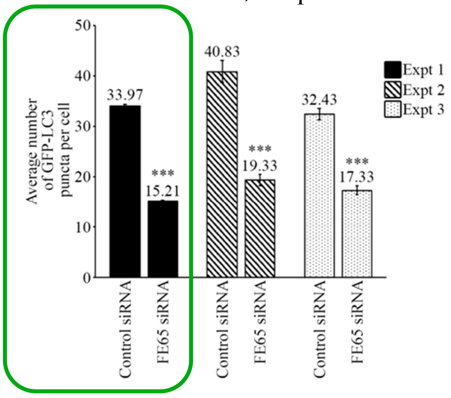

Figure S2. The Alphafold prediction was conducted using ChimeraX. Protein sequences of Beclin $1^{248-450}$, which encompass the entire ECD-BARA domain, and $FE65^{641-710}$, which include the C-terminal region, were utilized for the prediction. The resulting best model displays Beclin 1 as the purple chain and FE65 as the pink chain. ChimeraX identifies potential interactions by locating pairs of residues in close proximity (≤ 4.0 Å). Several bonds were noted, indicated by green dotted lines. Notably, the model predicts that Beclin 1 Gly386-Thr388 are in proximity to FE65 Arg684, and Beclin 1 Phe390 is near FE65 Ser680. These findings align with our experimental results, supporting the possibility of a direct interaction between FE65 and Beclin 1.

Best model:

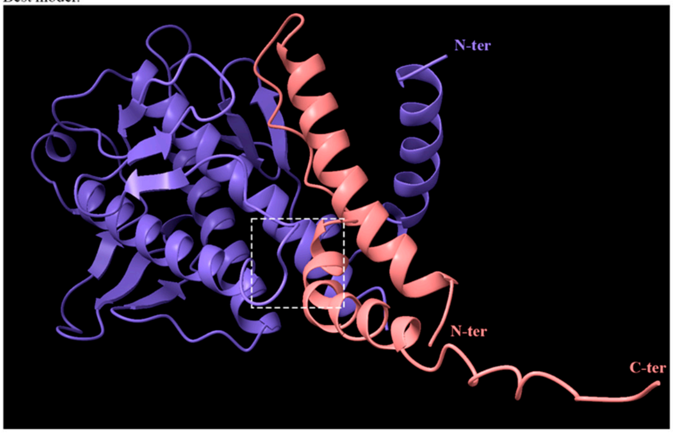

Predicted contact residues:

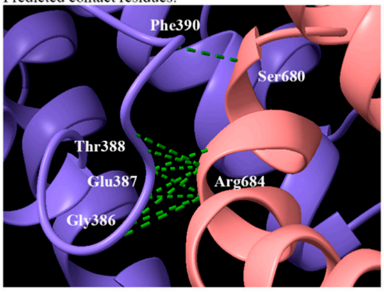

Figure S3. The quantification of GFP-LC3 puncta in GFP-LC3 stably expressing cells in the presence of FE65 or FE65$^{\Delta Ct}$ transfection, with and without Baf A1 treatment (Figure 3B). The experiments were performed in three independent replicates. In each experiment, at least 40 cells were analyzed per transfection. Data from experiment 1 (boxed in green) was presented in Figure 3B. Error bars are SEM, *** $p < 0.001$, ** $p < 0.01$.

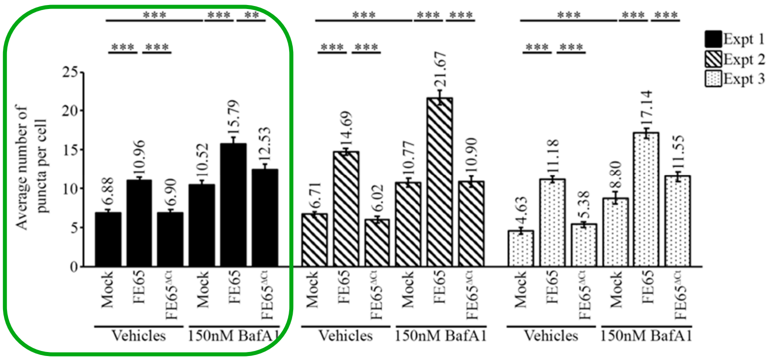

Figure S4. The quantification of WIPI-2 puncta in WT HEK293 and FE65 KO cells, with and without EBSS treatment (Figure 4C). The experiments were performed in three independent replicates. In each experiment, at least 40 cells were analyzed per group. Data from experiment 1 (boxed in green) was presented in Figure 4C. Error bars are SEM, *** $p < 0.001$, ** $p < 0.01$, * $p < 0.05$.

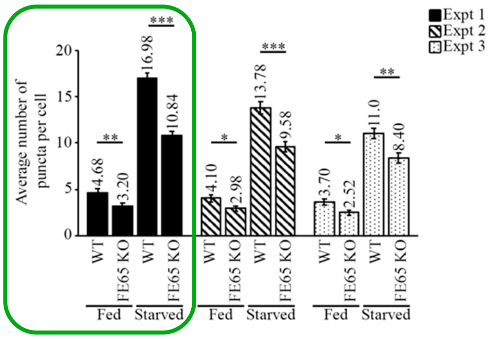

Figure S5. The quantification of WIPI-2 puncta in WT HEK293 in the presence of FE65 or FE65$^{\Delta Ct}$ transfection, with and without EBSS treatment (Figure 4D). The experiments were performed in three independent replicates. In each experiment, at least 40 cells were analyzed per transfection. Data from experiment 1 (boxed in green) was presented in Figure 4D. Error bars are SEM, *** $p < 0.001$, ** $p < 0.01$, * $p < 0.05$.

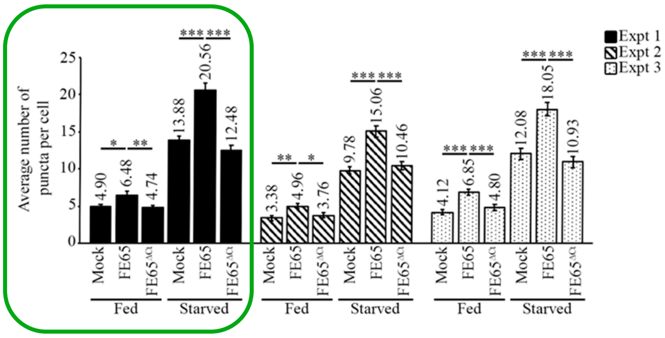

Figure S6. Biological replicate images of the co-IP experiments presented in Figure 2.
(A) Co-IP replicate experiments of FE65 and Beclin 1, as shown in Figure 2B (upper panel). **(B)** Co-IP replicate experiments of FE65 with members of the PI3KC3-C1 complex (ATG14L, Beclin 1, VPS34, and VPS15), as shown in Figure 2B (lower panel). **(C)** Co-IP replicate experiments conducted using rat brain lysate, as detailed in Figure 2C. **(D)** Co-IP experiments involving FE65, FE65 deletion mutants, and Beclin 1, as shown in Figure 2F. **(E)** Co-IP experiments of FE65, FE65$^{\Delta Ct}$, and Beclin 1, as depicted in Figure 2G.

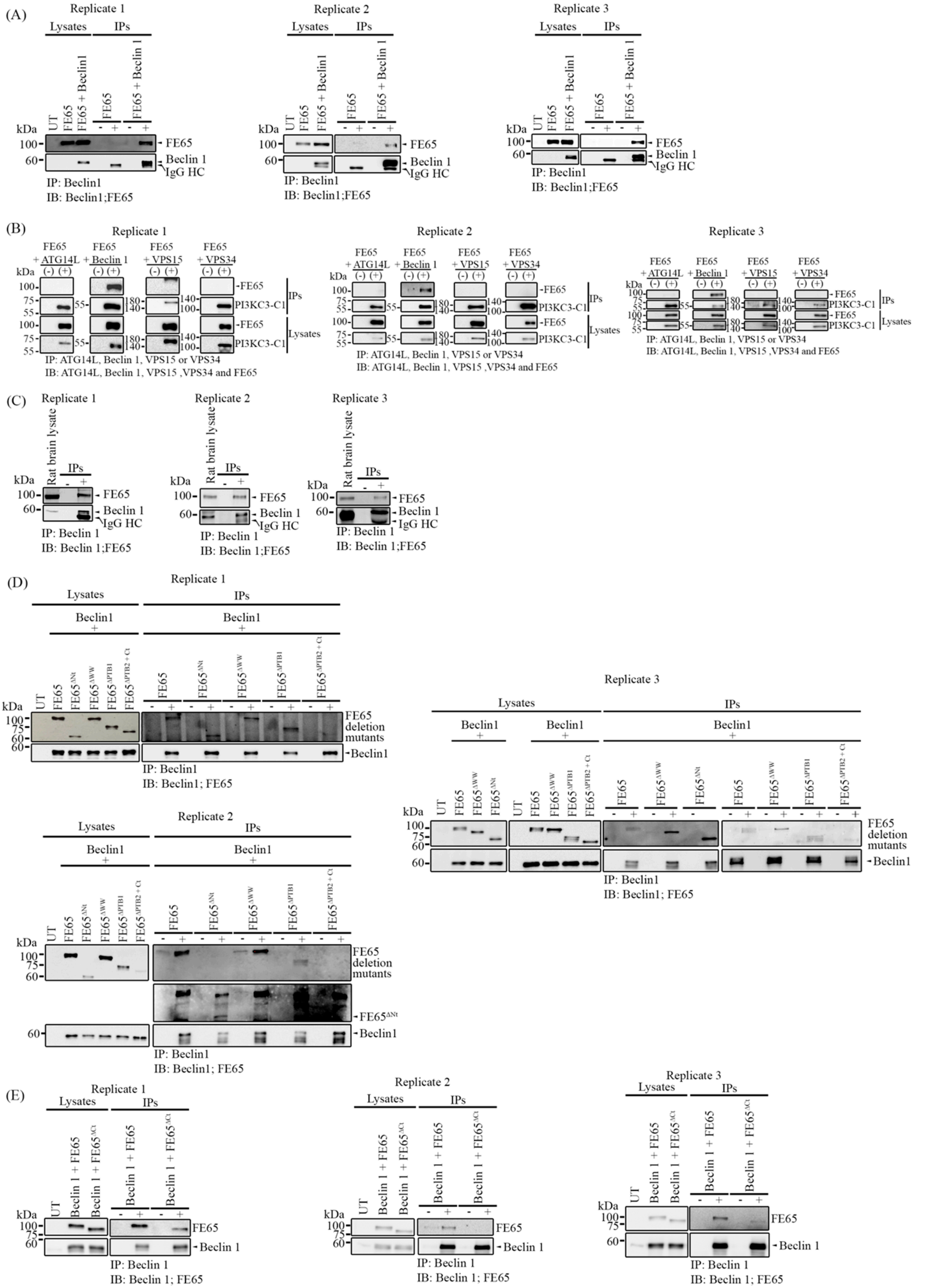

Figure S7. Biological replicate images of the p62 turnover experiments.
(A) Replicate experiments of p62 turnover in FE65 stable cells, as shown in Figure 1E. **(B)** Replicate experiments of p62 turnover in FE65 KO cells, as shown in Figure 1F. **(C)** Replicate experiments of p62 turnover in FE65 and FE65$^{\Delta Ct}$ stable cells, as shown in Figure 3C. **(D)** Replicate of the rescue experiments as illustrated in Figure 3E.

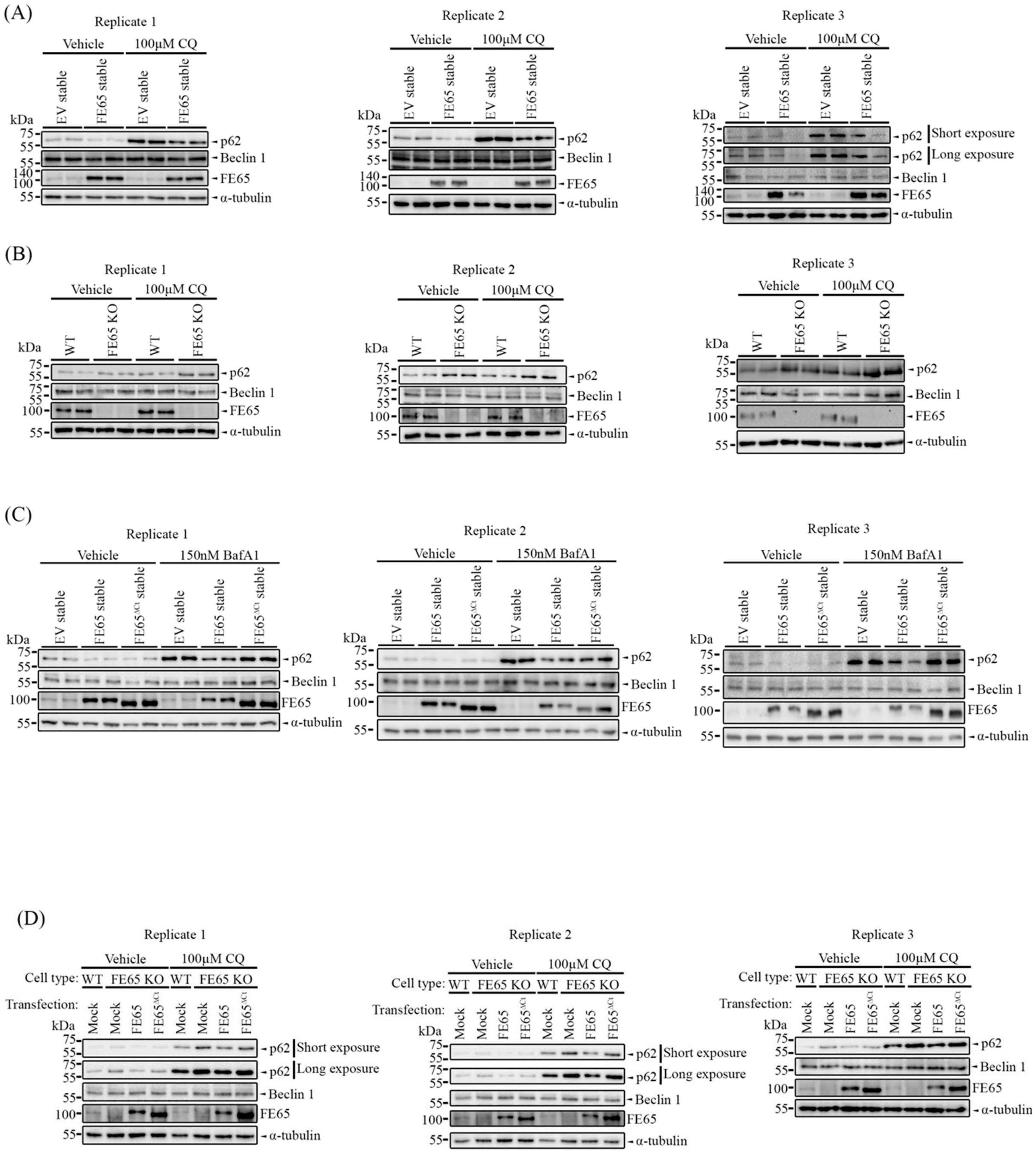

Figure S8. Enlarged immunofluorescence images presented in Figure 2D, 4C and 4D.
(A) Immunofluorescence images from the PLA experiment presented in Figure 2D.

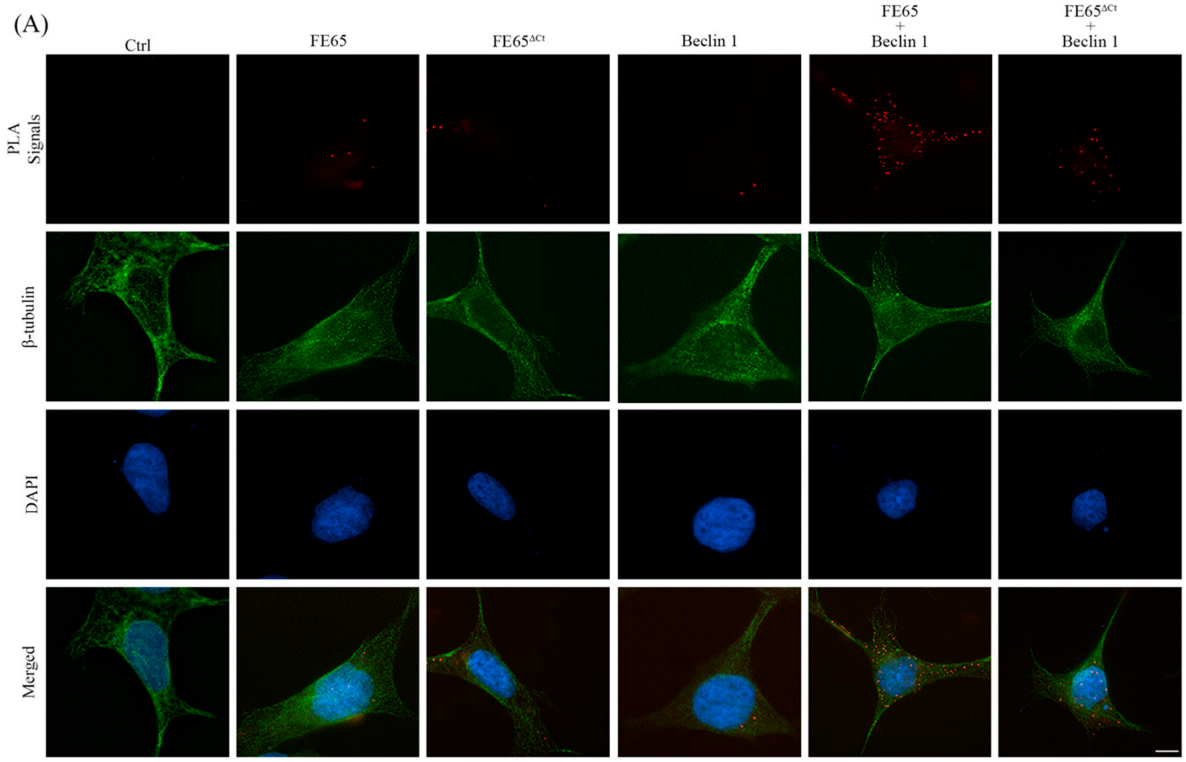

Figure S8. Enlarged immunofluorescence images presented in Figure 2D, 4C and 4D (con't)
(B) Immunofluorescence images of the WIPI-2 puncta presented in Figure 4C. **(C)** Enlarged immunofluorescence images of the WIPI-2 puncta presented in Figure 4D.

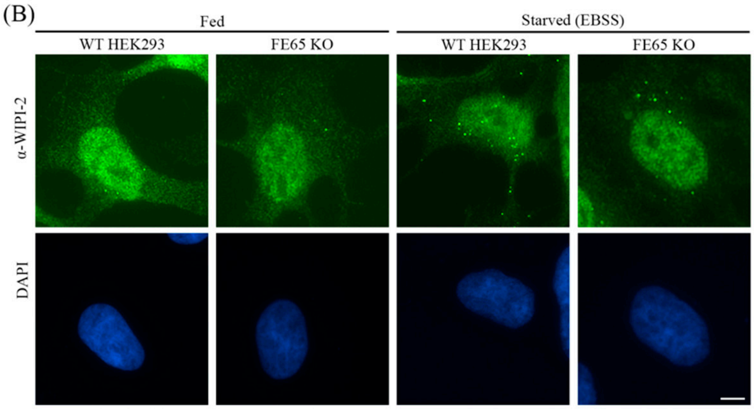

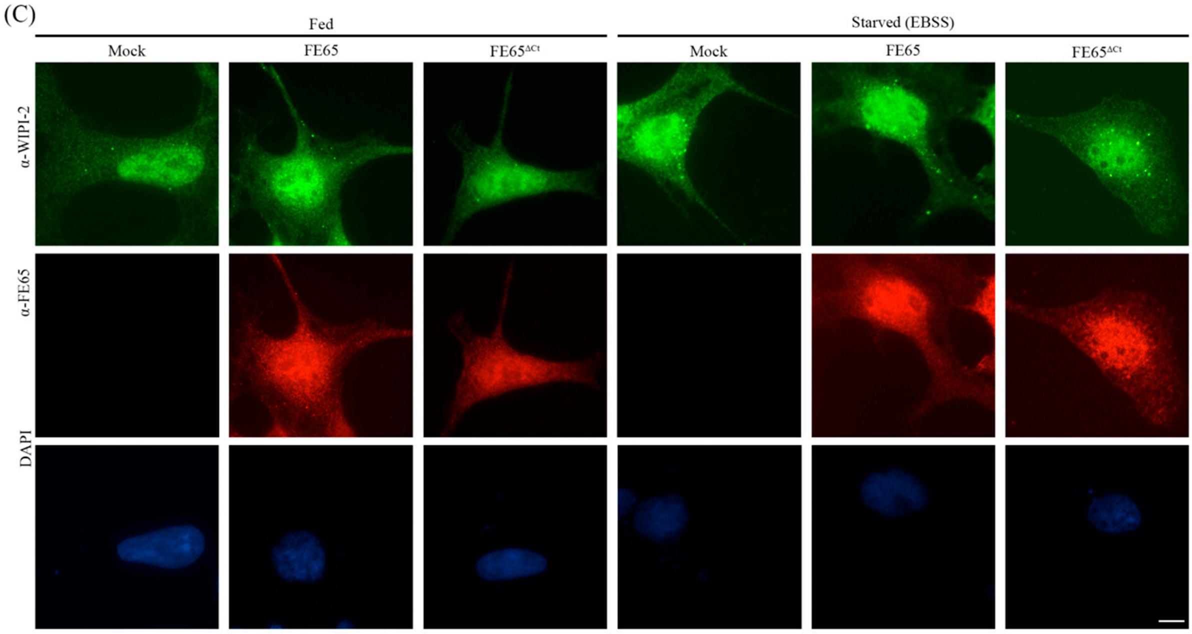

Supplement: Supplementary file 1 [file biology-14-00097-s001.zip › biology-3249966-supplementary.pdf]
